# Growth in Medicare Advantage by organizational size across rural and urban counties

**DOI:** 10.1111/jrh.70134

**Published:** 2026-03-30

**Authors:** Dan M Shane, Fred Ullrich, Keith J. Mueller

**Affiliations:** ^1^ Department of Health Management and Policy College of Public Health University of Iowa Iowa City Iowa USA

**Keywords:** medicare, medicare advantage, private insurance markets

## Abstract

**Purpose:**

To understand the changing landscape of Medicare Advantage (MA) markets in urban and rural counties with particular focus on the size of MA organizations. With MA covering more than half of all Medicare enrollees and notably different health and access conditions in rural areas, understanding the extent of participation in MA markets in both urban and rural areas is critical. In this paper, we establish which organizations are offering MA plans and which organizations’ plans are being chosen by eligible Medicare beneficiaries.

**Methods:**

Data on MA plan availability, parent organizations, and enrollment for 2019 through 2026 were obtained from CMS websites. Classification of counties was based on 2024 Urban Influence Codes (UIC): metropolitan (1, 4); micropolitan (2, 5, 7); nonmetropolitan (3, 6, 8, 9). We offer a taxonomy of MA organizations based on the number of states where MA organizations offer plans: national (29–50 states), large regional (6–18 states), small regional (2–5 states), local (1 state).

**Findings:**

The overall size of the MA population grew 59% from 2019 to 2025, including doubling growth in the most rural counties. The proportion of the MA population enrolled in plans from national MA organizations and small regional organizations has increased monotonically over this period. Notably, this trend has featured across both urban and rural counties. Enrollment in plans from local MA organizations has declined across all geographic locations.

**Conclusion:**

National MA organizations continue to dominate the landscape across both urban and rural counties, with nearly 65% of total enrollment in 2025. Moreover, over 80% of the growth in available plans from 2019 to 2025 were offered by these same four or five organizations.

## INTRODUCTION

As of 2023, Medicare Advantage (MA) plans cover more than half of all enrolled Medicare beneficiaries nationally.^1^ MA penetration increased 75% between 2014 and 2024, from 31% to 54% of enrolled beneficiaries. Moreover, while there were no states with greater than 50% MA enrollment in 2014, now half of all US states have a majority of eligible Medicare beneficiaries enrolled in an MA plan.^1^ Compared to original Medicare, MA plans typically cover additional services, such as prescription drugs or dental services, while featuring a narrower network of providers for enrollees.^2^ As Medicare Advantage continues to grow, these tradeoffs and implications for access to care may have a bigger impact on rural beneficiaries than on their urban counterparts. Rural areas suffer from a higher prevalence of chronic conditions and lower availability of clinical care, in addition to worse measures compared to urban counties on most other social determinants of health.^3^ Recent work using the Medicare Current Beneficiary Survey finds that rural enrollees in Medicare Advantage plans faced more barriers in accessing health services due to cost and rural Medicare enrollees overall were less likely to receive preventive services compared to urban enrollees.^4^ MA plans also feature utilization management strategies, including more frequent prior authorizations, claim denials, and potential bargaining leverage with respect to rural hospitals. Evidence on increasing MA enrollment on rural hospital finances is mixed. Henke et al.^5^ find that MA growth improved financial conditions for hospitals in areas with higher uninsured rates. Cataife and Liu,^6^ in contrast, find that higher MA penetration reduces paid inpatient days for rural hospitals, increasing financial distress. Shifts in bargaining power points to the potential for market concentration to become a more important policy issue, especially in smaller rural markets.

In typical insurance markets, more competition is associated with lower premiums.^7^ Plan premiums in Medicare Advantage, however, depend on the carrier's bid to cover services for Medicare Parts A and B compared to the administratively determined benchmark and thus the relationship between competition and premiums is less clear. If a plan's bid is below the benchmark, the MA premium for enrollees is typically zero (enrollees still would owe Part B and possibly Part D premiums) and the plan receives a rebate of the difference between bid and benchmark.^8^ These rebates must be passed through to enrolled beneficiaries either as additional benefits or payment reductions (potentially reducing Part B premiums, for example) and thus contribute to the additional benefits MA plans frequently offer. Recent work found that MA markets with fewer firms operating were associated with both higher premiums and higher quality ratings.^9^ In terms of the organizations offering plans, MA enrollment nationally skews heavily toward the largest organizations with the two largest firms covering almost 50% of all MA enrollees.^1^ Recent work by Hnath and co‐authors^10^ found that national carriers have expanded their market share in the last decade while regional carriers have seen a decline in market share.

The goal of this study is to add depth to our understanding of the competitive landscape in Medicare. We establish which organizations are offering MA plans and which organizations’ plans are being chosen by eligible Medicare beneficiaries. We further evaluate changes in both plan offerings and plan enrollment since 2019 and offer evidence on geographic differences in these trends across the United States, with particular attention to rural counties. We offer a taxonomy of MA organizations based on the number of states where they offer plans. We summarize changes in MA participation based on our organizational taxonomy over the last five years by metropolitan, micropolitan, and rural counties.

## BACKGROUND

### Data and methods

Data on MA plan availability, parent organizations, and enrollment for 2019 through 2026 were obtained from files downloaded from publicly available CMS websites.^11^, ^12^, ^13^ All data are provided at the county (or equivalent) level. Classification of counties was based on 2024 Urban Influence Codes (UIC): metropolitan (1, 4); micropolitan (2, 5, 7); nonmetropolitan (3, 6, 8, 9).^14^

MA plans were not available in 151 counties in 2019 and 116 counties in 2026. There were no plans available in the state of Alaska in either year. Connecticut was excluded from this report because of inconsistent FIPS code reporting in the MA and UIC data.*

**Table:** 

**Parent Organization—**A company that owns a controlling interest in one or more legal entities that have contracted with CMS to offer Medicare health benefits and/or prescription drug coverage.
**Plans—**A set of Medicare health benefits and/or prescription drug coverage offered by an organization that has contracted with CMS.^15^

## FINDINGS

Table 1 shows that the overall number of MA parent organizations and plans grew between 2019 and 2023 but started shrinking after that. Growth in MA plans continued until 2024 in micropolitan and rural counties before declining in the most recent years. The overall number of MA parent organizations and plans in counties was highest in metropolitan counties and lowest in rural counties.

**TABLE 1:** MA organization and plan counts, 2019–2026.

	Parent organizations				Plans			
	Total	Metro	Micro	Rural	Total	Metro	Micro	Rural
2019	131	130	89	71	2613	2574	1244	1032
2020	137	136	96	78	3012	2968	1469	1252
2021	139	138	97	83	3419	3361	1747	1497
2022	144	143	104	87	3688	3636	2022	1727
2023	142	141	105	88	3922	3863	2194	1963
2024	135	134	102	84	3879	3822	2245	2000
2025	123	123	93	78	3654	3621	2163	1924
2026	116	116	87	70	3324	3297	1973	1734

*Note*: Unweighted plan counts. Includes plans offered in 50 states and DC. Excludes SNPs, EGHPs, HCPPs, PACE plans, cost plans, and MMPs.

*Source*: CMS MA Landscape and Plan Directory Files 2019–2024.

Between 2019 and 2023, the number of parent organizations offering MA plans in metropolitan counties grew by 8.5% while those in micropolitan counties grew by 18.0% and those in rural counties grew by 23.9%. But since that time, the number of organizations offering MA plans has shrunk 17.7% in metropolitan counties, 17.1% in micropolitan counties, and 20.5% in rural counties. The number of plans those organizations have offered follows a similar pattern with growth between 2019 and 2023 of 50.0% in metropolitan counties, 76.4% in micropolitan counties, and 90.2% in rural counties. Since 2023, plan offerings have shrunk 14.7% in metropolitan counties, 10.0% in micropolitan counties, and 11.7% in rural counties.

The MA market nationally is dominated by a few large parent organizations, with UnitedHealthcare accounting for the highest percentage of MA enrollees (29% as of 2024).^1^ UnitedHealthcare offer plans in 48 states and DC and Humana offered plans in 46 states and DC in 2026. There are three additional organizations with a significant national footprint in MA plan offerings. In 2026, CVS Health Corporation offers MA plans in 42 states, Centene Corporation offers MA plans in 30 states, and Health Care Service Corporation (a Blue Cross and Blue Shield Association licensee) offered MA plans in 30 states. The remaining MA organizations have much smaller geographic footprints. We classified those organizations using the taxonomy shown in Table 2. There was a clear break in the number of states served between the smallest national organization (30 states) and the biggest large regional organization (21 states). The distribution of organizations by number of states where they offer plans also suggested a convenient break of 5–6 states for small and large regional organizations. Regulatory/statutory requirements (e.g. certification by state insurance regulators) suggest that there is also a significant organizational difference between those operating in a single state and those offering plans in two or more states.

**TABLE 2:** MA organization counts by size, 2019–2026.

	National (30–50 states)		Large regional (6–21 states)		Small regional (2–5 states)		Local (1 state)	
	Orgs.	% of counties	Orgs.	% of counties	Orgs.	% of counties	Orgs.	% of counties
2019	3	88.6%	9	64.7%	25	23.0%	94	40.7%
2020	3	89.4%	9	76.2%	30	26.3%	95	40.4%
2021	5	96.0%	8	42.5%	31	33.7%	95	44.9%
2022	5	97.2%	9	52.2%	36	49.6%	94	46.2%
2023	5	97.9%	10	56.4%	36	60.0%	91	40.4%
2024	4	96.1%	9	56.7%	40	67.7%	82	41.7%
2025	5	95.5%	6	47.4%	38	57.6%	74	40.8%
2026	5	92.8%	5	46.7%	31	42.0%	75	39.3%

The table shows the number of national organizations has been stable since 2021. Meanwhile, the number of large (plans in 6–21 states) and small regional organizations (plans in 2–5 states) has slowly decreased since 2022. Similarly, the number of local organizations (only offering plans in 1 state) held steady from 2019 to 2022 and then declined starting in 2023. Little of the shrinkage in the number of local organizations can be attributed to them shifting into a larger size category—one of the local organizations in 2025 transitioned to the small regional category in 2026. Five of the local organizations disappeared entirely while two new organizations appeared, and five organizations downsized from the small regional to the local category.

Table 2 also displays the proportion of counties between 2019 and 2026 with at least one plan offered by MA organizations using our size taxonomy. Overall, the proportion of counties with MA plans from national organizations steadily increased until 2023 and has declined since then. In 2026, plans from UnitedHealth Group, Inc. were available in 82.6% of all counties and plans from Humana Inc. were available in 84.5% of all counties (data not shown). The availability of MA plans from large regional organizations declined since 2019. The proportion of counties with plans from small regional organizations grew dramatically from 2019–2024 but has since declined. The proportion of counties with plans from local organizations was stable from 2019–2026.

Table 3 displays the average number of MA plans available in counties and the proportion of county MA enrollees from 2019–2026 based on offering organization size and geography. Overall, the average number of available plans more than doubled between 2019 (12.6 plans) and 2026 (26.4 plans). That same basic pattern exists across geographies, although the average number of plans available in rural counties overall grew approximately 134.9% between 2019 (8.6 plans) and 2026 (20.2 plans). Much of the growth in available plans was driven by increased offerings from national organizations (172.5% overall between 2019 and 2026) with even more pronounced growth in micropolitan (185.5%) and rural (200.0%) counties. Growth in available plans was also higher among the small regional MA organizations across all geographies. It should be noted that all of these growth rates have slowed since 2024.

**TABLE 3:** Mean number of plans available and percent MA enrolled^a^ in counties, by MA organization size, 2019–2026.

	Total		National (30–50 states)		Large regional (6–21 states)		Small regional (2–5 states)		Local (1 state)	
	Avg. plans	MA enrolled	Avg. plans	% MA enrolled	Avg. plans	% MA enrolled	Avg. plans	% MA enrolled	Avg. plans	% MA enrolled
**Overall**										
2019	12.6	20.8M	6.9	56.4%	2.1	18.9%	1.1	5.8%	2.4	18.9%
2020	15.2	22.9M	8.0	57.1%	3.3	19.2%	1.4	6.0%	2.5	17.7%
2021	19.3	25.3M	12.7	61.9%	2.2	15.6%	1.9	9.0%	2.5	13.5%
2022	23.8	27.6M	15.4	62.5%	2.8	15.6%	2.9	10.1%	2.7	11.8%
2023	28.1	29.8M	18.2	63.5%	3.6	15.7%	3.7	10.2%	2.6	10.6%
2024	28.8	31.9M	18.3	63.9%	3.4	15.5%	4.7	11.0%	2.4	9.5%
2025	28.8	33.1M	19.6	64.7%	2.7	13.0%	3.9	12.0%	2.5	10.2%
2026^b^	26.4	—	18.8	—	2.5	—	2.6	—	2.3	—
**Metropolitan**										
2019	17.7	18.2M	9.3	55.1%	3.3	20.4%	1.5	5.7%	3.6	18.9%
2020	21.3	19.9M	10.7	55.8%	5.0	20.5%	2.0	5.9%	3.7	17.8%
2021	26.5	21.8M	16.7	60.6%	3.5	16.8%	2.7	8.9%	3.6	13.7%
2022	31.4	23.6M	19.5	61.2%	4.3	16.8%	3.8	10.1%	3.9	11.9%
2023	36.2	25.3M	22.3	62.1%	5.6	17.0%	4.6	9.9%	3.7	10.9%
2024	36.8	27.0M	22.4	62.6%	5.3	16.8%	5.6	10.9%	3.4	9.7%
2025	36.7	28.1M	24.4	63.8%	4.0	13.9%	4.9	11.8%	3.4	10.6%
2026^b^	34.2	—	23.7	—	3.5	—	3.7	—	3.1	—
**Micropolitan**										
2019	11.7	1.6M	6.2	63.4%	1.8	9.5%	1.3	7.5%	2.4	19.6%
2020	14.2	1.8M	7.5	64.2%	2.9	11.0%	1.4	7.2%	2.4	17.6%
2021	18.1	2.1M	11.9	68.4%	1.8	8.4%	2.0	9.8%	2.4	13.4%
2022	22.9	2.4M	15.0	69.0%	2.4	8.6%	2.9	10.4%	2.6	12.0%
2023	27.4	2.7M	17.9	69.8%	3.1	8.5%	3.8	12.0%	2.6	9.7%
2024	27.8	2.9M	17.8	69.6%	2.8	8.7%	4.8	12.5%	2.4	9.3%
2025	27.4	3.0M	18.6	69.1%	2.4	7.9%	4.2	14.4%	2.3	8.6%
2026^b^	24.7	—	17.7	—	2.2	—	2.6	—	2.0	—
**Rural**										
2019	8.6	1.0M	5.0	68.3%	1.2	7.6%	0.7	6.1%	1.5	18.0%
2020	10.2	1.2M	5.9	67.9%	2.0	9.8%	0.8	5.9%	1.5	16.4%
2021	13.4	1.4M	9.5	71.5%	1.2	8.0%	1.1	9.4%	1.5	11.1%
2022	17.2	1.6M	11.8	72.1%	1.6	8.4%	2.1	9.1%	1.6	10.4%
2023	21.1	1.8M	14.6	73.1%	2.1	8.2%	2.9	10.7%	1.6	8.0%
2024	22.1	2.0M	14.8	73.2%	1.9	8.3%	3.8	10.9%	1.6	7.6%
2025	22.2	2.0M	15.7	72.0%	1.8	8.0%	2.9	12.1%	1.7	7.9%
2026^b^	20.2	—	15.0	—	1.6	—	1.8	—	1.6	—

*Note*: CMS censors county/plan data where 10 or fewer beneficiaries are enrolled. The data presents undercounted totals.

*Source*: CMS MA Landscape and Plan Directory Files 2019–2024 and State/County/Contract enrollment files 2019–2024.

^a^ Excludes Cost, MMP, and PACE plans.

^b^ Plan enrollment data is not yet available for 2026.

As reported elsewhere, the overall size of the MA population has grown significantly since 2019. During that time, the proportion of the population enrolled in plans from national organizations and in plans from small regional organizations has grown monotonically. At the same time, enrollment in plans from large regional organizations has decreased everywhere except in rural counties. Enrollment in plans from local organizations has decreased across all geographic locations.

## DISCUSSION

Availability of MA plans continues to grow in the United States as nearly 3300 plans are available nationwide and over 1700 plans are now available across rural counties. It is unclear to what extent the number of MA plans available to a typical Medicare‐eligible resident reflects a competitive market. We contribute to this important question in three ways: (1) by establishing a consistent taxonomy for categorizing MA organizations based on the number of states where they offer plans, (2) creating a snapshot of plan availability and plan enrollment across rural and urban counties using this taxonomy, (3) contrasting evidence from 2019 to understand key trends. National organizations (operating in 30–50 states) continue to dominate the landscape with more than 60% of all MA enrollment across all geographic regions. Three quarters of the growth in the number of plans available (on average) in a given county were additional plans offered by these largest carriers. Further, more than 80% of the growth in MA enrollment between 2019 and 2025 is with national organizations, a pattern we find across both rural and urban counties. There has also been a notable rise in the share of enrollment among small regional organizations offering plans in between two and five states. Coincident with the rise in small regional enrollment has been a drop in the enrollment share for MA organizations offering plans in only one state (local). These shifts in MA organizations responsible for the growth in plans and enrollment have implications for consumers and health care organizations. Hospitals, particularly those in rural counties with historically lower MA penetration rates, may experience challenges with utilization management strategies and negotiating with national MA organizations compared to local or regional organizations, particularly if only one national organization is present in the county. For enrollees in rural areas, the tradeoffs of additional benefits from an MA plan versus a narrower network of providers differ from urban enrollees, particularly as rural areas suffer from a higher prevalence of chronic conditions and suffer from more acute care provider shortages. Important follow‐up research will aim to evaluate differences in utilization of services across rural and urban MA enrollees as well as differences in claim denials and prior authorizations using our newly developed taxonomy. Understanding the value of MA plans and potential differences depending on the size of offering MA organizations is an important next step. For policy makers considering adjustments to Medicare payments to MA organizations, understanding patterns depending on MA organization size and geographic footprint is an important consideration. Following the trends uncovered in this paper with additional evidence after any changes in payments or changes in utilization management practices for MA plans is also an important next research step.

## CONFLICT OF INTEREST STATEMENT

The authors declare no conflicts of interest.

## ETHICS STATEMENT

De‐identified secondary data were used for this research and thus was exempt from IRB review

## Footnotes

* In 2022, the US Census Bureau formally recognized Connecticut's Councils of Government (COG) as county equivalents. During the period of the data in this report, CMS reported Connecticut Medicare enrollment by county until 2022 when it switched to reporting by COG. The available data does not support converting counts between the two types of geography. Therefore, Connecticut data has been excluded from this report.
